# Gender disparities in out-of-hospital cardiac arrest: implications for public health and bystander interventions in Zhengzhou

**DOI:** 10.3389/fmed.2025.1681676

**Published:** 2026-01-07

**Authors:** Xiaopeng Liu, Xiaoqing Yang, Yifan Huang, Xiaozhan Yang, Hongjiang Zhang, Shaojiang Lv, Xiuzhen Kong, Hongyu Wang, Sisen Zhang

**Affiliations:** 1 Department of Emergency Medicine, The Fifth Clinical Medical College of Henan University of Chinese Medicine, Zhengzhou, Henan, China; 2 Henan Cardiopulmonary and Brain Resuscitation Engineering Technology Research Center, Zhengzhou, Henan, China; 3 Department of Emergency Medicine, Huaxian People’s Hospital, Anyang, Henan, China; 4 Department of Emergency Medicine, Zhongmu County People’s Hospital, Zhengzhou, Henan, China; 5 Department of Emergency Medicine, People’s Hospital of Henan University of Chinese Medicine/People’s Hospital of Zhengzhou, Zhengzhou, Henan, China

**Keywords:** out-of-hospital cardiac arrest, gender disparities, cardiopulmonary resuscitation, shockable rhythm, return of spontaneous circulation

## Abstract

**Background:**

Out-of-hospital cardiac arrest (OHCA) remains a critical public health crisis in China, with survival rates below 1%, notably lower than those reported in developed nations (10%–12%). Gender disparities have emerged as crucial determinants of OHCA outcomes; however, China-specific evidence remains limited.

**Methods:**

We conducted a retrospective cohort study of 3,272 adult non-traumatic OHCA patients treated at the Zhengzhou Emergency Medical Rescue Center between 2017 and 2020. Patient characteristics and interventions were analyzed using multivariable logistic regression to assess their associations with prehospital return of spontaneous circulation (ROSC).

**Results:**

Among 3,272 OHCA patients, women accounted for 27.6% (*n* = 902), and were significantly older than men (70.1 vs. 61.1 years, *p* < 0.001). Compared with men, women experienced lower rates of witnessed arrest (51.2% vs. 64.0%, *p* < 0.001), CPR from bystanders (4.8% vs. 7.3%, *p* = 0.01), AED use (8.9% vs. 12.4%, *p* = 0.005), and shockable initial rhythm (6.7% vs. 16.1%, *p* < 0.001). In addition, women were less likely to experience an OHCA in a public location (13.7% vs. 26.4%, *p* < 0.001). The female sex remained independently associated with a significantly lower prehospital ROSC rate (OR: 0.26; 95% CI: 0.13–0.53, *p* < 0.001).

**Conclusion:**

Significant gender disparities were identified in prehospital outcomes of OHCA patients in Zhengzhou. Compared with men, women were older and less likely to experience a cardiac arrest in public locations, have the event witnessed, receive cardiopulmonary resuscitation (CPR) from a bystander, present with a shockable rhythm, or require an automated external defibrillator (AED). These factors collectively contributed to substantially lowered prehospital ROSC rates among women. Targeted public health strategies–such as expanding inclusive CPR training, promoting gender-sensitive AED use, enhancing community awareness of female resuscitation, and optimizing emergency medical service protocols–are urgently required to reduce these disparities.

## 1 Introduction

Cardiac arrest is an acute and fatal condition that poses a significant threat to public health. In China, the estimated incidence of out-of-hospital cardiac arrest (OHCA) is approximately 40 per 100,000 person-years, with annual cases exceeding 550,000 (1). In the United States, the incidence is about 110 per 100,000 person-years (approximately 350,000 cases annually), but the survival rates are markedly higher at 10%–12% (2). This discrepancy highlights the urgent need for improved resuscitation systems and public health interventions in China.

Epidemiological studies worldwide have reported on consistent gender-based disparities in OHCA outcomes. Compared to women, men are more likely to experience witnessed cardiac arrests, receive cardiopulmonary resuscitation (CPR) from bystanders, present with shockable rhythms, and ultimately achieve better survival rates (3).

Numerous studies have identified key predictors of OHCA outcomes, which include age, cardiac arrest location (public vs. private), whether the event was witnessed, CPR was administered by bystanders, initial cardiac rhythm, emergency medical service (EMS) response time, and automated external defibrillator (AED) use–collectively known as Utstein variables (4–9). However, cultural and social factors often discourage the public from performing CPR on women due to fears of inappropriate contact, potential accusations of sexual assault, or the risk of causing injury (10). These barriers may contribute to observed gender disparities in OHCA outcomes (11–15). Beyond social influences, biological and physiological differences between the sexes may also affect outcomes following an OHCA. Additionally, several studies have reported that women are typically older at the time of cardiac arrest, more likely to experience an OHCA at home, and less likely to present with a shockable rhythm compared to men (13, 14). These differences are thought to contribute significantly to poorer survival outcomes among women. Although some investigators have suggested that sex hormones could result in biological effects, most available evidence emphasizes disparities in resuscitation characteristics and system-level responses rather than the hormonal status between men and women. These findings support the hypothesis that gender plays a critical role in OHCA incidence and outcomes.

Despite the growing evidence on sex-based differences in OHCA outcomes globally, there is a notable lack of corresponding data from China. A recent national survey showed that public CPR training rates remained below 1% (1), and most existing studies did not examine sex-specific disparities. This knowledge gap limits the development of targeted strategies for improving resuscitation efforts across genders. To address this issue, our study investigated gender disparities in OHCA characteristics and prehospital outcomes in Zhengzhou, China. Using a large EMS-based dataset, we analyzed key prognostic variables–such as initial rhythm, bystander CPR, and AED use–through a gender-sensitive lens. Our findings aim to inform localized public health interventions and EMS protocols, while contributing to the global discourse on sex-based inequalities in emergency care.

## 2 Materials and methods

### 2.1 Study design and setting

This retrospective observational cohort study was conducted using EMS records from the Zhengzhou Emergency Medical Rescue Center (ZEMRC), the sole emergency medical service provider in Zhengzhou, Henan Province, serving a population of over 10 million across 60 EMS stations and 7 regional sub-center-satellite emergency dispatch units, and supporting field operations and coordinating with ambulance services across designated zones. The study was performed in accordance with strengthening the reporting of observational studies in epidemiology (STROBE) guidelines for observational studies.

### 2.2 Ethical approval

This study was conducted in accordance with the ethical standards outlined in the Declaration of Helsinki and was approved by the Medical Ethics Committee of Zhengzhou People’s Hospital (Approval No. ZZRMYYLL033). All data were anonymized before analysis. Given the retrospective nature of the study and absence of personally identifiable information, the requirement for written informed consent was waived by the ethics committee.

### 2.3 Study population

Eligible patients included adults aged 18 years or older who experienced non-traumatic OHCA and received resuscitative efforts from EMS at ZEMRC, Zhengzhou, Henan province, China, between July 1, 2017 and June 30, 2020. We excluded cases where resuscitation was not attempted due to do-not-resuscitate (DNR) orders, obvious signs of death (e.g., rigor mortis, decapitation), or missing demographic data required for survival analysis. All data were anonymized before analysis.

### 2.4 Data collection and variable definitions

Clinical and resuscitative variables were extracted from standardized EMS case report forms based on the 2020 revised version of the Utstein template for out-of-hospital cardiac arrest reporting (16). Data regarding the following variables were collected: age, sex, arrest location (public vs. private), witnessed status, bystander CPR administration (yes/no), AED use, initial cardiac rhythm (shockable vs. non-shockable), epinephrine administration, tracheal intubation, and EMS response time (in minutes). The EMS response time was defined in accordance with Utstein standards as the interval in minutes between the receipt of the emergency call at the dispatch center and the arrival of the first EMS crew at the patient’s side. The cardiac arrest location was categorized as either “public” or “non-public” in accordance with the Utstein template. A public location was defined as any site, including streets, highways, public transportation stations, parks, markets, shopping centers, schools, and workplaces, which was accessible to the public. Non-public locations included private residences, nursing homes, and other restricted-access facilities. “AED use” was defined as the administration of defibrillation using a public access AED before EMS arrival.

### 2.5 Outcome measurements

The primary outcome was the pre-hospital ROSC, defined as the restoration of a palpable pulse and effective circulation sustained until arrival at the emergency department, as documented by EMS personnel. This definition was in accordance with Utstein reporting guidelines. No secondary outcomes were evaluated in this study. In-hospital or post-discharge survival data were unavailable from EMS records; therefore, survival was not included as an outcome measure.

### 2.6 Statistical analysis

All statistical analyses were performed using SPSS software (version 25.0, IBM Corp., Armonk, NY). Descriptive statistics were used to summarize the baseline characteristics of the study population. Categorical variables were reported as counts and percentages, and compared using Chi-square or Fisher’s exact tests, as appropriate. Continuous variables were presented as means with standard deviations (SD) or medians with interquartile ranges (IQR), and compared using independent sample *t*-tests or Mann-Whitney U tests, depending on data distribution.

Multivariable logistic regression models were constructed for inferential analysis to evaluate the association between gender and the primary outcome, i.e., ROSC. Covariates were selected based on clinical relevance, data availability, and prior evidence from the literature and the Utstein reporting framework. Specifically, the following variables were included in the multivariable logistic regression model: age, sex, cardiac arrest location (public vs. private), witnessed status, bystander CPR, AED use, initial cardiac rhythm (shockable vs. non-shockable), tracheal intubation, and EMS response time. These factors are widely recognized as key predictors of OHCA outcomes and are consistent with internationally accepted Utstein variables (11, 13, 16). Model calibration was assessed using the Hosmer–Lemeshow test. Multicollinearity among covariates was evaluated using variance inflation factors (VIFs), with a VIF < 2 considered acceptable.

Additionally, we performed logistic regression analyses to determine predictors of survival across the entire cohort and within specific subgroups. As part of a secondary analysis, we examined factors associated with a shockable initial rhythm using a similar modeling strategy, with adjustments for resuscitation characteristics and age.

Missing data were handled through case exclusion. The proportion of missing values for each variable and total number of excluded patients are reported in the Section “3 Results.” A two-sided *p*-value of <0.05 was considered statistically significant.

## 3 Results

Of the 3,561 EMS-attended OHCA cases initially identified between July 2017 and June 2020, 289 patients (8.1%) were excluded due to missing key data on sex, ROSC status, or Utstein variables. The final analysis included 3,272 patients. The proportions of missing data were as follows: age (0.2%), arrest location (0.7%), witnessed status (1.5%), bystander CPR (2.1%), AED use (1.9%), initial rhythm (2.3%), and EMS response time (0.6%).

This study analyzed 3,272 EMS-treated OHCA patients, including 902 (27.6%) women and 2,370 (72.4%) men. A total of 1979 cases (60.1%) were witnessed by bystanders, and 215 (6.6%) received CPR from bystanders. Initial shockable rhythms were observed in 441 patients (13.5%). Cardiac arrests occurred in household settings in 2,409 cases (73.6%), while 749 (22.9%) occurred at public locations.

The mean age of female patients was significantly higher than that of males (70.1 vs. 61.1 years, *p* < 0.001). Table 1 shows that most OHCAs occurred at private locations (77.1%), and the median EMS response time was 8.0 min for both genders.

**TABLE 1:** Characteristics and Utstein predictors of out-of-hospital cardiac arrest patients by gender.

Variable	Total	Gender		*P*-value
		Men	Women	
*N* (%)	3272 (100)	2370 (72.4)	902 (27.6)	
Age (years), mean (SD)	63.7 (17.0)	61.1 (16.7)	70.1 (15.8)	<0.001
OHCA at a public location, *n* (%)	749 (22.9)	625 (26.4)	124 (13.7)	<0.001
Witnessed OHCA, *n* (%)	1979 (60.1)	1517 (64.0)	462 (51.2)	<0.001
Bystander CPR, *n* (%)	215 (6.6)	172 (7.3)	43 (4.8)	0.01
Shockable initial rhythm, *n* (%)	441 (13.5)	381 (16.1)	60 (6.7)	<0.001
AED use *n* (%)	373 (11.4)	293 (12.4)	80 (8.9)	0.005
Median EMS response time interval (IQR)	8.0 (4.5)	8.0 (4.5)	8.0 (4.5)	0.816
Tracheal intubation, *n* (%)	912 (27.9)	680 (28.7)	232 (25.7)	0.09
Epinephrine, *n* (%)	3245 (99.2)	2349 (99.1)	896 (99.3)	0.533
ROSC, *n* (%)	190 (5.8)	177 (7.5)	13 (1.4)	<0.001

AED, automated external defibrillator; CPR, cardiopulmonary resuscitation; EMS, emergency medical services; IQR, interquartile range; OHCA, out-of-hospital cardiac arrest; ROSC, return of spontaneous circulation; SD, standard deviation.

**FIGURE 1:**
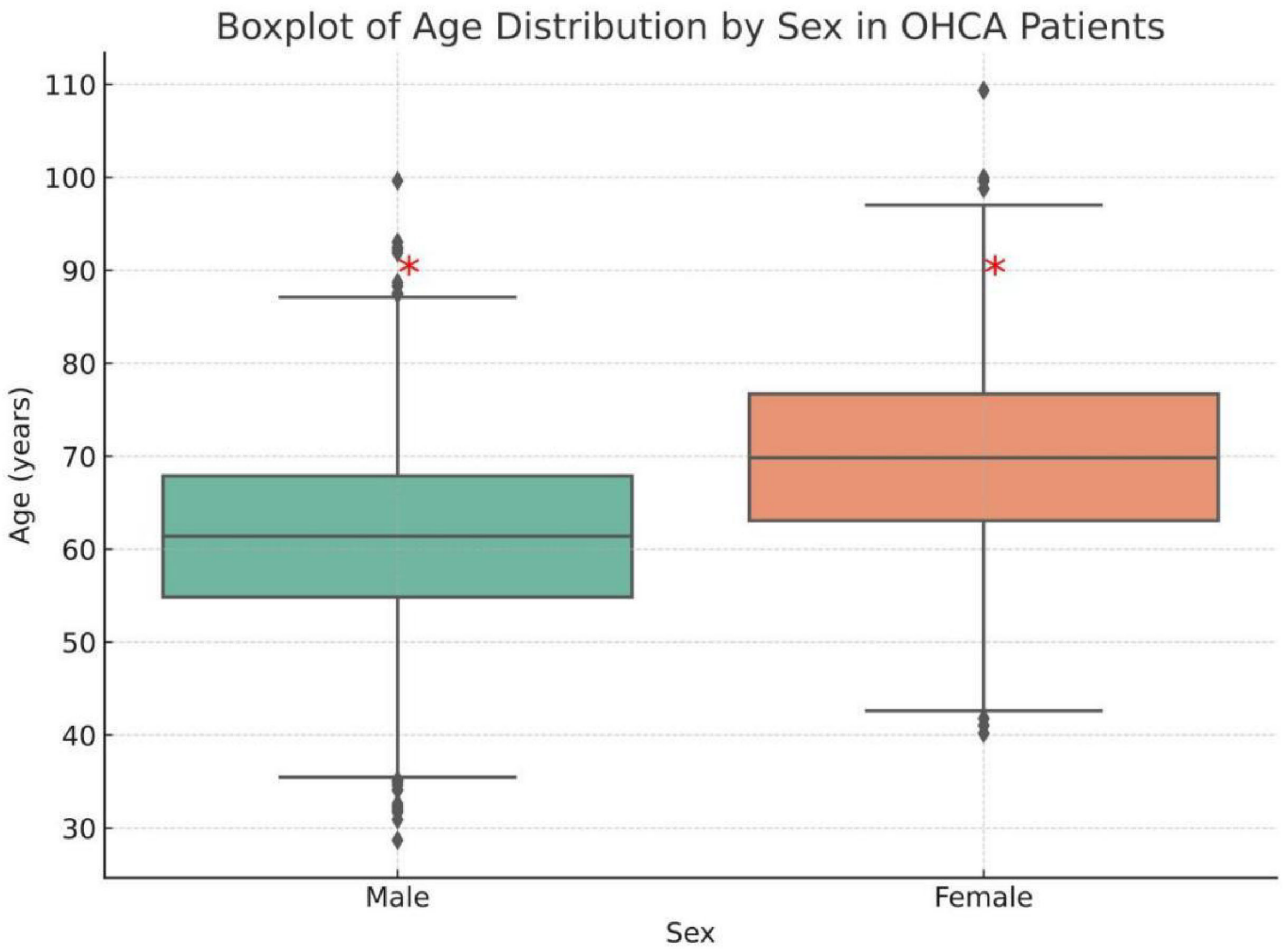
Boxplot of age distribution in male and female patients experiencing an out-of-hospital cardiac arrest (OHCA). The red asterisk (*) indicates a statistically significant difference between male and female groups (*p* < 0.05).

**FIGURE 2:**
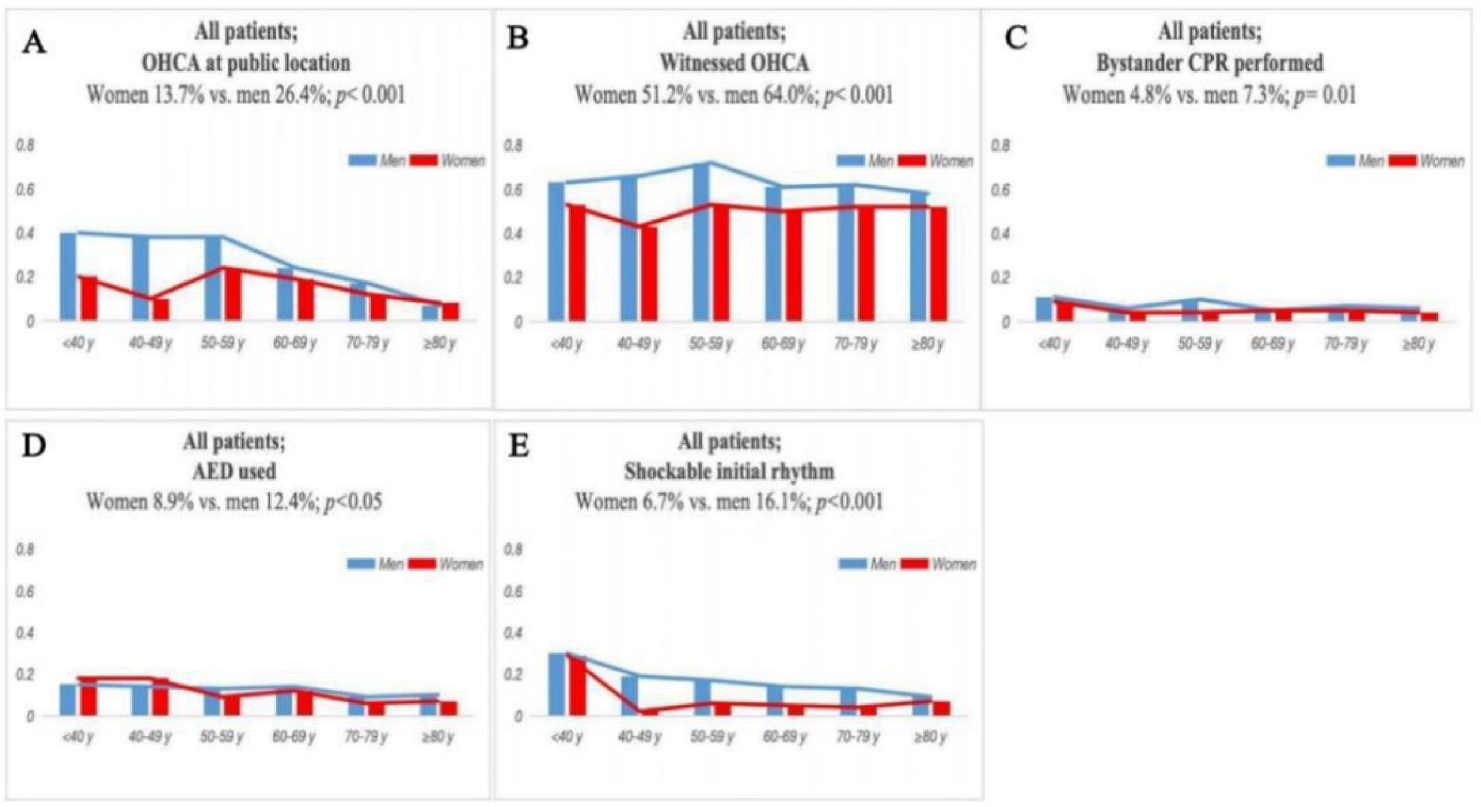
Comparison of OHCA-related variables between male and female patients across different age groups. Compared variables include: **(A)** OHCA at public location, **(B)** witnessed OHCA, **(C)** bystander CPR performed, **(D)** AED used, and **(E)** shockable initial rhythm.

Significant gender-associated disparities were observed across Utstein predictors. Compared to men, women were less likely to have their OHCA witnessed (51.2% vs. 64.0%, *p* < 0.001), receive CPR from bystanders (4.8% vs. 7.3%, *p* = 0.01), or present with a shockable initial rhythm (6.7% vs. 16.1%, *p* < 0.001). AED use was also lower in women (8.9% vs. 12.4%, *p* = 0.005). Figure 1 displays the age distribution of out-of-hospital cardiac arrest (OHCA) patients by sex. The median age among female patients was higher than that of male patients (approximately 70 vs. 61 years), and the interquartile range was wider among women, indicating a broader age distribution. Additionally, female patients demonstrated more high-end outliers, suggesting a higher proportion of elderly women in the OHCA population. As shown in Figure 2, age-stratified analysis revealed consistent gender disparities across all age groups, particularly in the receipt of bystander CPR and the presence of a shockable initial rhythm. The difference in shockable rhythm was more pronounced in younger patients, while older women demonstrated lower rates of AED use and witnessed arrest compared to men in the same age category.

Table 2 presents the results of multivariable logistic regression for ROSC. Gender remained a significant independent predictor of ROSC after adjusting for confounders. The female sex was associated with decreased odds of achieving ROSC (OR: 0.26, 95% CI: 0.13–0.53, *p* < 0.001). Other significant predictors of ROSC included a younger age (OR per year increase: 0.90, 95% CI: 0.88–0.91), OHCA occurrence in public locations (OR: 1.66, 95% CI: 1.07–2.57, *p* = 0.024), bystander CPR (OR: 2.43, 95% CI: 1.42–4.12, *p* = 0.001), AED use (OR: 4.48, 95% CI: 2.82–7.13, *p* < 0.001), and a shockable initial rhythm (OR: 2.77, 95% CI: 1.87–4.11, *p* < 0.001). The multivariable logistic regression model demonstrated acceptable calibration (Hosmer–Lemeshow *p* = 0.44). All covariates had VIFs < 1.8, indicating no multicollinearity-associated concerns.

**TABLE 2:** The adjusted multivariable logistic regression model for ROSC.

Variable	Adjusted odds ratio (95% CI)	*P*-value
Women	0.26 (0.13–0.53)	<0.001
Age	0.90 (0.88–0.91)	<0.001
OHCA at public locations	1.66 (1.07–2.57)	0.024
Shockable initial rhythm	2.77 (1.87–4.11)	<0.001
Witnessed OHCA	1.02 (0.62–1.68)	0.928
Bystander CPR	2.43 (1.42–4.12)	0.001
AED use	4.48 (2.82–7.13)	<0.001
Tracheal intubation	1.26 (0.86–1.86)	0.246
EMS response time interval	0.98 (0.93–1.02)	0.299

AED, automated external defibrillator; CPR, cardiopulmonary resuscitation; EMS, emergency medical services; OHCA, out-of-hospital cardiac arrest.

Further analysis of Table 3 revealed the factors associated with the presence of a shockable initial rhythm. Women were significantly less likely to present with a shockable rhythm than men (OR: 0.52, 95% CI: 0.39–0.70, *p* < 0.001), even after adjusting for age, location, and bystander CPR. Younger age, public location, and receipt of bystander-administered CPR were positively associated with the presence of a shockable rhythm. In univariate analysis, older age, female sex, non-public location, and non-shockable rhythm were significantly associated with lower ROSC rates (all *p* < 0.05).

**TABLE 3:** Adjusted multivariable logistic regression model for shockable initial rhythm.

Variable	Adjusted odds ratio (95% CI)	*P*-value
Women	0.52 (0.39–0.70)	<0.001
Age	0.98 (0.97–0.98)	<0.001
OHCA at public locations	1.39 (1.05–1.74)	0.021
Bystander CPR	2.86 (2.05–3.98)	<0.001

CPR, cardiopulmonary resuscitation; OHCA, out-of-hospital cardiac arrest. Within the cohort, 441 patients (13.5%) presented with a shockable initial rhythm.

## 4 Discussion

This study provides novel evidence on gender disparities in OHCA outcomes in Zhengzhou using a large, real-world EMS database. In contrast to earlier studies from Western countries or national registries, our work highlights a pronounced gap in bystander CPR and AED use among women–particularly in a setting with low public CPR training penetration.

First, it demonstrates that women in Zhengzhou who experience OHCA are significantly less likely to receive bystander CPR and AED interventions. This disparity may be attributable to sociocultural barriers, such as discomfort with performing chest compressions on female victims in public, as reported in both Chinese and international studies. Second, despite comparable cardiac arrest characteristics, women presented with significantly fewer shockable rhythms, suggesting delayed recognition or suboptimal efforts at resuscitation.

Our findings are consistent with those of prior studies showing that women with out-of-hospital cardiac arrest (OHCA) are generally older, have lower rates of shockable rhythms, and receive defibrillation less frequently than men (17–19). Several factors may contribute to these differences. First, women may experience delays in recognition and bystander intervention. Cultural and social concerns, such as discomfort with performing chest compressions on female patients, are reported to act as potential barriers to timely resuscitation (20). Second, physiological and etiological differences may be relevant. Women have a higher likelihood of experiencing OHCA resulting from non-cardiac causes, such as respiratory or metabolic events, and are less likely to present with shockable rhythms (21). Additionally, the advanced age of female OHCA patients may reflect a later onset of cardiac disease and could be associated with reduced baseline cardiac function. However, our dataset lacked cause-specific classification or cardiac function indicators, limiting our ability to verify these mechanisms. Despite the presence of AEDs in some public areas, bystander defibrillation is performed infrequently in China due to low public awareness and training. Lower defibrillation rates in females are probably attributable to a reduced incidence of shockable rhythms, although public hesitancy might partially contribute to this phenomenon (22–24). These observations underscore the importance of tailored public education efforts, gender-inclusive CPR training models, and expanded AED deployment in community settings.

The quality of CPR was worse in women than in men, and the results of univariate and multivariate logistic regression analyses showed gender associated differences in ROSC rates. Some researchers have proposed that estrogen has cardioprotective effects, potentially delaying the onset of coronary artery disease (CAD) in women. This may help explain why female OHCA patients are typically older than men at the time of cardiac arrest (20, 21). However, once cardiac arrest occurs, a higher age may be associated with a decline in baseline cardiac function and a lower likelihood of shockable rhythms, as observed in our data. In this context, the initial cardioprotective benefits of estrogen may be outweighed by age-related physiological decline at the time of cardiac arrest. Further research is needed to understand the interplay between sex hormones, age, and cardiac arrest outcomes.

Our results were consistent with those of certain previous studies, although some inconsistencies were noted in the results of previous studies. A previous study showed that CPR characteristics and ROSC rates were worse in women than in men (11). In contrast, another study showed that while ROSC rates were worse in women than in men in univariate analysis, no gender based differences in ROSC rates were observed after multivariate adjustment (13). Another study showed that ROSC was better in women than in men after multivariate adjustment (14). These findings may reflect the regional disparities in emergency response infrastructure and public health education. For example, in many urban areas of China, including the setting of our study, the availability of AEDs in public spaces remains limited, and public CPR training is not widespread. This may contribute to lower rates of bystander intervention and defibrillation compared to other countries. Additionally, differences in EMS deployment models and response times across regions may influence outcomes. These contextual factors highlight the importance of tailoring public health interventions to regional needs.

This study contributes several key findings that extend beyond biological hypotheses. First, it demonstrates that women in Zhengzhou who experience an OHCA are significantly less likely to receive bystander-administered CPR and AED interventions. This disparity may be influenced by sociocultural barriers such as discomfort with performing chest compressions on female victims in public, a phenomenon reported in both Chinese and international studies. The lower rate of shockable rhythms observed in female patients may be multifactorial. Some studies have suggested that delays in recognizing cardiac arrest symptoms or initiating resuscitation could reduce the likelihood of detecting a shockable rhythm at the time of EMS arrival (25). However, our dataset did not include time-to-recognition or CPR quality indicators; hence, we cannot determine whether these factors contributed to our population associated findings. Further research is needed to clarify the temporal dynamics between arrest detection, rhythm evolution, and outcomes across sexes.

Even after adjusting for key resuscitation variables, such as bystander CPR and AED use, female sex remained an independent negative predictor of prehospital ROSC in our multivariable model. Our findings are consistent with recent evidence highlighting complex interactions between age and sex in OHCA outcomes. Chen et al. (26) conducted a large multicenter analysis in an Asian EMS system and found that female patients received better prognoses than males in younger age groups, but this survival advantage was reversed in older patients. This observation supports the notion that the effect of sex on cardiac arrest outcomes is not uniform and may be modified by age and other contextual factors.

This suggests that sex-based disparities in OHCA outcomes may be influenced by a combination of biological, social, and contextual factors beyond public intervention. Possible contributors include differences in underlying etiology, baseline health status, EMS response, and unmeasured aspects of prehospital care. Further studies are warranted to explore these mechanisms in greater depth.

Additionally, although the selection of covariates for multivariable analysis was based on internationally accepted Utstein variables and supported by the findings of prior studies, it is notable that much of this evidence originates from studies focused on Western populations. Differences in EMS systems, cultural norms regarding bystander response, and healthcare accessibility may limit the direct applicability of these predictors in the Chinese context. Future studies using nationally representative OHCA registries in China are needed to validate and refine predictive models for local use.

Several limitations are associated with this study. First, as a retrospective observational study, it is subject to inherent selection bias and unmeasured confounders. Second, data on important factors, such as comorbidities, exact time intervals (e.g., collapse-to-CPR, response time), quality of bystander-associated CPR, and cause-specific cardiac arrest were not available, which limits the interpretation of causality. Third, our outcome was limited to prehospital ROSC rates; we did not assess sustained ROSC, survival to hospital discharge, or neurological outcomes, which are clinically more meaningful. Fourth, this was a single-center study conducted in an urban area of China, and the findings may not be applicable to rural settings or other healthcare systems. Finally, although we performed multivariable adjustment, residual confounding from unmeasured factors (such as EMS crew experience or hospital-level differences) cannot be excluded.

This study is based solely on EMS data from Zhengzhou and reflects the characteristics of a single urban setting. Therefore, the findings–particularly those regarding bystander behavior, AED access, and EMS infrastructure–may not be applicable to rural areas or other provinces in China, where cultural, economic, and medical systems differ considerably.

## 5 Conclusion

Significant gender disparities exist in the prehospital outcomes of OHCA patients in Zhengzhou. Compared with men, women were older and less likely to experience cardiac arrest in public locations, have the event witnessed, receive bystander-administered CPR, present with a shockable rhythm, or undergo AED use. These combined factors contributed to substantially lower prehospital ROSC rates among women. Targeted public health strategies, including expansion of inclusive CPR training, promotion of gender-sensitive AED use, enhancement of community awareness of female resuscitation, and optimization of EMS protocols, are urgently required to reduce these disparities.

## Data Availability

The raw data supporting the conclusions of this article will be made available by the authors, without undue reservation.
